# *Saccharina japonica* Ethanol Extract Ameliorates Dextran Sulfate Sodium-Induced Colitis via Reshaping Intestinal Microenvironment and Alleviating Inflammatory Response

**DOI:** 10.3390/foods12081671

**Published:** 2023-04-17

**Authors:** Kuan Lu, Lin Liu, Pengcheng Lin, Xiufang Dong, Laixue Ni, Hongxia Che, Wancui Xie

**Affiliations:** 1 College of Marine Science and Biological Engineering, Qingdao University of Science and Technology, Qingdao 266042, China; 2 Qingdao Keda Future Biotechnology Co., Ltd., Qingdao 266042, China; 3 Qingdao Special Food Research Institute, Qingdao 266109, China; 4 Linyi Jinluo Wenrui Food Co., Linyi 276007, China; 5 College of Food Science and Engineering, Qingdao Agricultural University, Qingdao 266109, China; 6 Shandong Provincial Key Laboratory of Biochemical Engineering, College of Marine Science and Biological Engineering, Qingdao University of Science and Technology, Qingdao 266042, China

**Keywords:** *Saccharina japonica*, inflammatory bowel disease, intestinal barrier, gut microbiota, short-chain fatty acids

## Abstract

*Saccharina japonica* belongs to brown macro-alga with various potential health benefits; its antioxidant and anti-inflammatory activities indicate the potential to improve inflammatory bowel diseases. Here, the potential anti-colitis effect of *Saccharina japonica* extract (SJE) was evaluated on dextran sulfate sodium (DSS)-induced ulcerative colitis (UC) in C57B/L6 mice. The mice were treated with mesalazine (MES) and various doses of SJE by gavage for 14 days. Results showed that both MES and SJE treatment decreased the disease activity index scores, relieving the short colon. SJE increased the occludin and zonula occludens-1 levels, and the beneficial effects were better than MES. MES and SJE exerted similar effects in decreasing inflammatory cytokines and oxidative stress. Moreover, SJE reshaped the intestinal microbiota by increasing α-diversity and reducing plenty of harmful bacteria. Dietary SJE was significant to relieving the reduction in short-chain fatty acids. The results revealed the protective effect of SJE on colitis and potential mechanisms, which is important for the rational use of SJE in UC prevention.

## 1. Introduction

Ulcerative colitis (UC) is a non-specific chronic colonic inflammatory disease widely prevalent in the world along with industrialization and urbanization [1]. Among them, unhealthy lifestyle (such as staying up late or overeating) is considered to be the main cause of UC [2], while intestinal mucosal barrier disruption, inflammatory response and intestinal microbiota dysbiosis are the main pathological features of UC [3]. Available studies have shown that patients with UC not only have persistent abdominal pain, diarrhea, blood in the stool and weight loss but also have a significantly increased risk of developing colon cancer [4]. Therefore, failure to find appropriate treatments to mitigate the effects of UC on patients will place a tremendous burden on their health, lives and the nation’s healthcare system [5]. However, the drugs currently used clinically to treat UC are usually anti-inflammatory and immunosuppressive drugs, and their long-term use can lead to serious side effects and cause severe physical and psychological damage to the human body [6]. Therefore, non-pharmacological UC remission has attracted widespread attention from patients; especially, the prevention and remission of UC by means of dietary and nutritional interventions is gradually becoming the top research spot. 

It has been described that in clinical studies, specific nutrients derived from food such as dietary fiber and curcumin alleviated UC [7,8]. Recently, the protective effects of crude extract and even full-nutrient foods on improving intestinal inflammation have been explored. For example, Ferramosca et al. (2019) [9] found that the extract of *Prunus mahaleb* fruit could improve colitis via regulating the Nrf2 pathway. Moreover, Han et al. (2019) [10] found a protective effect of the whole strawberry in inflammatory diseases of the intestine via maintaining the balance of the immune system and reshaping the structure of the intestinal microbiota. Notably, crude extract of Fuzhuan brick tea also showed a potential protective effect against colitis via regulating oxidative stress and inflammatory cytokines [11]. Furthermore, *Rhodiola crenulata* extract showed excellent beneficial effects on mice with colitis through inhibiting the secretion of inflammatory factors and maintaining the integrity of the intestinal barrier [5]. The improvement of UC by crude extracts has attracted widespread attention. Therefore, it is extremely important to discover more crude extracts to develop new nutritional interventions for different UC populations. However, most of the current studies have focused on crude extracts from terrestrial sources, with a few relevant reports on the ameliorative effects of crude extracts from marine sources on UC.

*Saccharina japonica* (SJ) belongs to brown macro-alga, which is generally produced in Asian countries, including China, Japan, and Korea [12]. It has a long history of use not only as a food but also as a medicine, and it is an important source of marine drugs. Dong et al. (2021) [13] found that Saccharina japonica extract (SJE) by the ethyl acetate phase was rich in fucoidan polyphenols, which have a strong ability to inhibit oxidase activity and scavenge free radicals. In addition, carbohydrates such as fucoidan polysaccharides contained in SJE can synergize with polyphenols to present better antioxidant activity [14]. Yu et al. (2023) [15] found that SJE contained a high concentration of fucoxanthin (121 mg/kg), which could alleviate symptoms such as obesity or tumors by up-regulating the expression level of coupling proteins and regulating the composition of intestinal microbes in addition to showing strong antioxidant activity. Increasingly, studies have shown that SJE are a rich source of anti-inflammatory compounds, and this bioactive principle ranges from polyphenols (approximately 10%), cellulose, polysaccharides, fatty acids, and alginic acid to carotenoids [16]. In addition, SJE has shown significant efficacy in the prevention and regulation of inflammation, oxidative stress and diabetes because of its strong antioxidant, anti-inflammatory and lipid metabolism-modulating biological effects [17,18,19]. Therefore, SJE is gradually considered as a new marine functional product that can effectively relieve the symptoms of UC. However, the effects of SJE on dextran sulfate sodium (DSS)-induced colitis remain unexplored and unclear. 

Therefore, the potential protective effects of SJE on colitis were explored. For that, several factors, including inflammatory responses, intestinal histopathological changes, gut barrier integrity, oxidative stress, microbiotacomposition and abundance, and metabolite short-chain fatty acids (SCFAs), were analyzed. This study provides a theoretical basis for the SJE to improve UC and nutritional effects, leading to several features that potentiate the SJE applicability.

## 2. Materials and Methods

### 2.1. Materials

*Saccharina japonica* purchased from Kaiping Farmers’ Market (Qingdao, Shandong Province, China) were used in the extraction process. DSS was purchased from MP Biomedicals LLC. (Irvine, CA, USA) with a molecular weight of 36,000–50,000 Da. Phloroglucinol (≥98%) was purchased from Solarbio Science and Technology Co., Ltd. (Beijing, China). Mesalazine (MES) was obtained from the Sunflower Pharmaceutical Group Co., Ltd. (Jiamusi, China). Enzyme-linked immunosorbent assay kits for the detection of interleukin (IL)-1β, IL-10, interferon-γ (IFN-γ), and tumor necrosis factor-α (TNF-α) were obtained from Dakewe Biotech Co., Ltd. (Shenzhen, China). An enhanced BCA protein assay kit, superoxide dismutase (SOD) assay kit with NBT, lipid peroxidation MDA assay kit, and TUNEL kit were purchased from Beyotime Biotechnology Co., Ltd. (Shanghai, China). A urine fecal occult blood test kit was bought from Nanjing Jiancheng Bioengineering Institute, Nanjing, China. Anti-mouse zonula occludens-1 (ZO-1) and occludin were purchased from ABclonal Technology (Wuhan, China). 

### 2.2. Preparation of SJE

The preparation method of SJE was performed according to Dong et al. (2021) [13] with slight modifications. In short, saccharin is ground into a powder using a mixer. Powdered saccharin (500 g) was extracted twice with 5 L of 85% (*v*/*v*) ethanol at 40 °C. After suction, the filtrate was concentrated in a vacuum rotary evaporator. The residue was stored overnight at 4 °C and filtered with suction to remove precipitated mannitol. Finally, SJE was obtained by lyophilization.

### 2.3. Animal Breeding and Intervention

The animal breeding and intervention procedures were adjusted according to the method developed by Dong et al. (2021) [13] with minor modifications. Male, 8-week-old C57BL/6 mice with body weight (BW) at 20–22 g were bought from Qingdao Daren Fortune Animal Technology Co., Ltd. (Qingdao, Shandong Province, China). The breeding conditions were: temperature 22–25 °C, humidity 55 ± 5%, and 12/12 h light/dark cycle. After a week of acclimation, all animals were randomly divided into six groups (8 mice for each): normal group, DSS group, MES group (10 mg/kg BW), low-dose SJE (L-SJE, 1 g/kg BW), medium-dose SJE (M-SJE, 2 g/kg BW), and high-dose SJE (H-SJE, 4 g/kg BW). 

**Figure 1:**
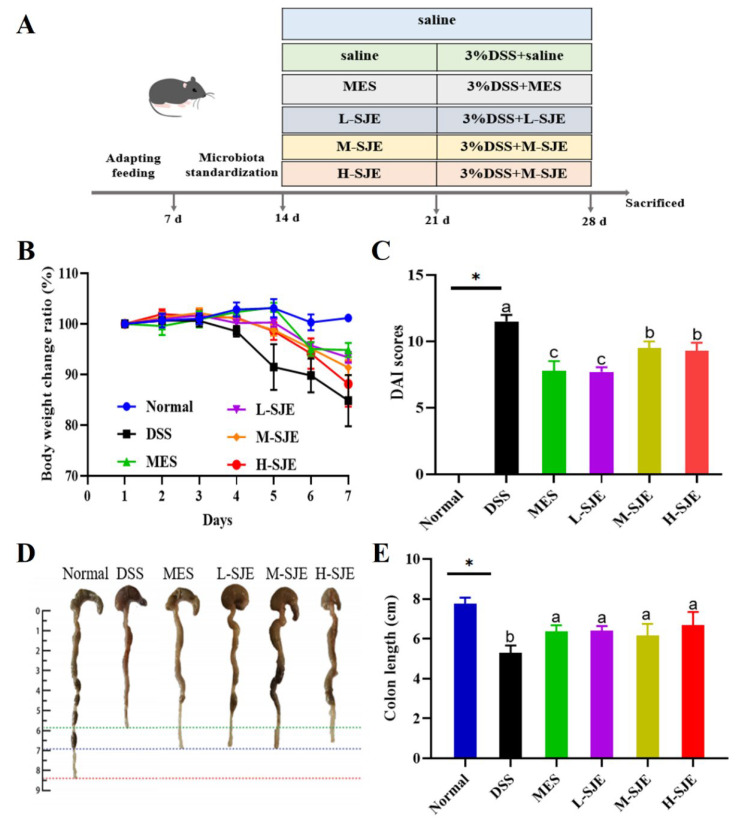
SJE intervention alleviated symptoms of colitis. (**A**) The information of experimental design, (**B**) body weight change ratio, (**C**) DAI score, (**D**) representative pictures of colons, (**E**) colon length. * *p* < 0.05 means statistically significant. Different superscript letters (a–c) in the same graph indicate significant differences between groups (*p* < 0.05).

The animals of normal and DSS groups were treated with saline, and the treated groups received MES and different doses of SJE from the 15th day to the 21st day. Then, all the mice, except the normal group, had free access to 3% DSS water for the last seven days. All the treated groups received different samples once a day. The experimental design is shown in Figure 1A. After fasting for 12 h on the 28th day, all animals were sacrificed by cervical dislocations under anesthesia. The design and conduction of whole experiments were strictly abided by the guide for animal welfare ethics. The study was approved by the Animal Ethics Committee of the College of Food Science and Engineering of Qingdao University of Science and Technology (Approval No. HYXY20191018). After the length measurement, the colons were cut into three parts. In addition, fresh feces from mice were collected. All tissues and feces were placed in liquid nitrogen for short-term storage and then stored at −80 °C.

### 2.4. Calculation of Disease Activity Index (DAI)

DAI was calculated according to our previous study [20]. The formula was: DAI = (bodyweight loss score + stool consistency score + gut bleeding score)/3.

### 2.5. Alcian Blue Staining and Hematoxylin and Eosin (HE) Staining

For alcian blue staining, the paraffin slides were dewaxed, and afterwards, these slides were stained in alcian blue dye solution A (for 15 min) and alcian blue dye solution B (for 3 min). Finally, the slides were washed with tap water. Changes in the colon tissue were observed and captured using a light microscope after dehydration. The colon tissue was embedded in paraffin, and 5 μm sections were cut for HE staining. Deparaffinization was conducted before staining with hematoxylin solution for 3–5 min. Finally, the slides were stained with eosin dye. The damage score was assessed according to inflammation extent, crypt status, lymphocyte infiltration extent, and colon wall abnormalities [21].

### 2.6. Transmission Electron Microscopy (TEM)

TEM was performed as previously described [22]. Briefly, after fixation, the collected tissues were dehydrated and embedded with resin. Then, 70 nm thin sections were cut on the ultra-microtome (Leica, Wetzlar, Germany). Finally, the slides were placed in a solution containing a uranium acetate saturated alcohol solution and lead citrate. TEM (H-7650, Hitachi, Tokyo, Japan) was used to obtain images.

### 2.7. Immunohistochemistry (IHC)

The IHC experiment was conducted according to our previous study [23]. First, the collected colon tissues were embedded in paraffin. Then, the tissues were deparaffinized and rehydrated. After this, the tissues were subjected to antigen retrieval and blocking. Then, the samples were incubated with the primary antibody against ZO-1 and occludin at 4 °C overnight. After washing with PBS 1X (three times), the tissues were stained with 3,3-diamino-benzidine-tetrahydrochloride (DAB) with HRP-labeled secondary antibody. All the samples were observed with a light microscope (E100, Nikon, Tokyo, Japan) and imaging system (Nikon DS-U3, Nikon, Japan). The image analysis system (Image-pro Plus 6.0, Media Cybermetics, Rockville, MD, USA) investigated for positive area and intensity. We performed immunohistochemical analysis. IHC score = P (pi × i) = (percentage of weak intensity area × 1) + (percentage of moderate intensity area × 2) + (percentage of strong intensity area × 3). pi: percentage of pixel area of positive signal; i: positive level. The larger the H-score value, the stronger the comprehensive positive intensity.

### 2.8. TdT-Mediated dUTP Nick-End Labeling (TUNEL) Assay Detection

Apoptotic colonic cells were detected using a TUNEL assay kit (Roche, Indianapolis, IN, USA) according to the manufacturer’s instructions, and images were captured with a microscope (Olympus, Tokyo, Japan) The proportion of positive cells in each tissue was calculated under 3 visual fields. The apoptotic index (percentage of apoptotic nuclei) = (apoptotic nuclei/total nuclei) × 100%.

### 2.9. Assessment of Oxidative Stress and Inflammation Response in Colon

The assessment of oxidative stress and inflammation response in the colon were performed according to the method developed by Chen et al. (2019) [22] with minor modifications. Briefly, the colon was homogenized in a saline solution (1:9 *w/v*) on ice. The homogenized sample was centrifuged (10,000× *g*, 4 °C ) for 15 min. The total protein concentration of the collected supernatant was measured using an enhanced BCA protein assay kit. Then, SOD activity and MDA level were measured using commercial kits. Furthermore, the IL-1β, TNF-α, IFN-γ, and IL-10 levels were detected using ELISA kits, and to measure the optical density, a microplate reader 680 (Bio-Rad, Hercules, CA, USA) was used.

### 2.10. Feces Collection and 16S rRNA Sequencing Analysis

The fresh feces were placed into a sterilized centrifuge tube (1.5 mL) and transferred to −80 °C for storage. The E.Z.N.A. stool DNA kit (Omega, Norcross, GA, USA) was used to extract total DNA, and a nanodrop was used to quantify the DNA. After that, 1.2% agarose gel electrophoresis was used to detect the DNA integrity. The V3–V4 region of 16S rRNA was amplified. The PCR amplification products were subjected to fluorescence quantification using a fluorescence reagent, Quant-iT PicoGreen dsDNA assay kit. The fluorescence was quantified using a microplate reader (BioTek, FLx800, Winooski, VT, USA). The validated library was used for sequencing on HiSeq2500 (Illumina, San Diego, CA, USA) and generating 2 × 300 bp paired-end reads.

### 2.11. Content Detection of SCFAs

The SCFAs analysis was performed according to the method developed by Dong et al. (2021) [13] with minor modifications. Briefly, phosphoric acid solution (0.5% *v/v*) was added to the 20 mg fecal sample. Then, the sample was vortexed for 10 min and sonicated for 5 min. Afterwards, the sample was centrifuged at 12,000 r/min for 10 min at 42 °C, and 100 μL of the supernatant was recovered and added to a new tube. Then, 300 μL of methyl tert-butyl ether solvent was added to the sample and vortexed for 3 min. Finally, repeat centrifugation, and the supernatant was analyzed by gas chromatography (GC).

### 2.12. Statistical Analysis

Data are presented as mean ± standard deviation (SD). The SPSS software (version 19.0, SPSS Inc., Chicago, IL, USA) was used for the statistical analysis. One-factor analysis of variance (ANOVA) and Tukey’s post hoc test were used to obtain the significant difference between DSS, MES, and SJE groups. Data were assumed to be statistically significant when *p* < 0.05. Different letters indicate significant differences between the groups. Origin (OriginLab Corporation, Northampton, MA, USA) was used for line chart analysis, bar chart analysis, principal component analysis (PCA), and box plot analysis. 

## 3. Results

### 3.1. SJE Alleviates Clinical Symptoms of Colitis

The changes in the BW were measured, and the results are presented in Figure 1B. The results demonstrated that the administration of SJE significantly inhibited the weight loss caused by DSS. Furthermore, both MES and SJE significant inhibited the increase in the DAI score induced by DSS. Interestingly, MES and L-SJE exerted similar effects, since a decrease in the DAI scores was observed. Additionally, the improvement of L-SJE was significant to that of M-SJE and H-SJE (Figure 1C). Furthermore, the results also demonstrated that the DSS treatment significantly reduced the colon length, and the MES and SJE treatments significantly inhibited the decrease in colon length (Figure 1D,E).

### 3.2. Effect of SJE on Histopathological Changes of Colon

**Figure 2:**
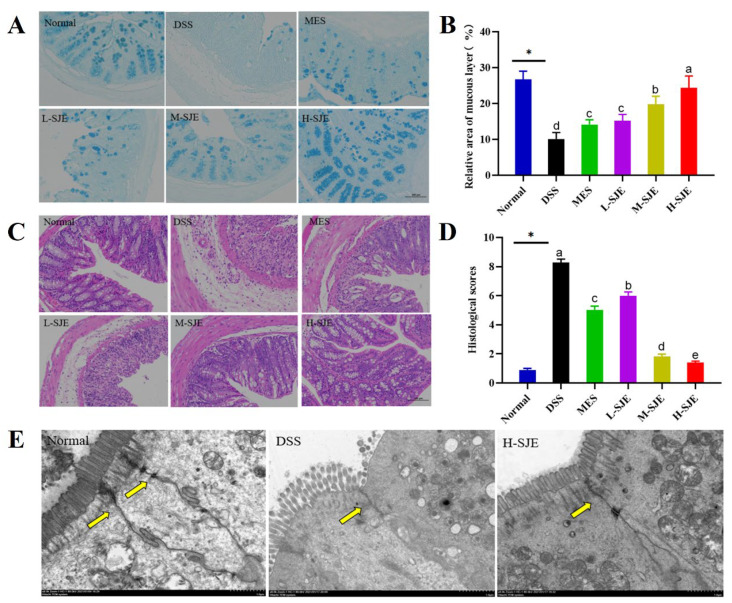
Effects of SJE on histopathological changes. (**A**) Results of alcian blue staining, (**B**) Alcian blue staining scores, (**C**) Representative picture of HE staining, (**D**) Histological scores, (**E**) Representative picture of TEM. * *p* < 0.05 means statistically significant. Different superscript letters (a–e) in the same graph indicate significant differences between groups (*p* < 0.05).

The histopathological changes of the colon are shown in Figure 2A. A large number of goblet cells were found in the normal group, which were evenly distributed and arranged neatly. After DSS treatment, a reduction in the goblet cells and mucus was observed. Furthermore, both MES and SJE treatments increased the area of the mucous layer. Interestingly, SJE treatment significant increased the number of goblet cells and promoted mucus secretion in a dose-dependent manner. In addition, the beneficial effect of H-SJE was the most significant, as shown in Figure 2A,B. 

The results of HE staining are shown in Figure 2C,D, and significant endothelial cell damage and higher histology score in the DSS group were observed, including the loss of crypts and leukocyte infiltration. The observed damage was significantly reduced after SJE treatment. Consistent with the results of alcian blue staining, a better outcome was obtained with H-SJE treatment. The results revealed an intact intercellular ultrastructure in the normal group (Figure 2E). Moreover, the intestinal mucosal villi were intact, neatly, and tightly arranged, and the length was uniform. Compared to the normal group, the intestinal mucosal villi of the model group are incomplete, loosely arranged, and varying in length. It is impossible distinguish tight junctions, adhesive junctions, and desmosomes clearly. The structure of the organelles was blurred, and the number decreased. In the H-SJE group, the intestinal mucosal villi were intact and uniform. The structure of tight junctions, adhesive junctions, and desmosomes was clear. In addition, the organelles were neatly arranged.

### 3.3. SJE Improves the Gut Barrier Integrity

**Figure 3:**
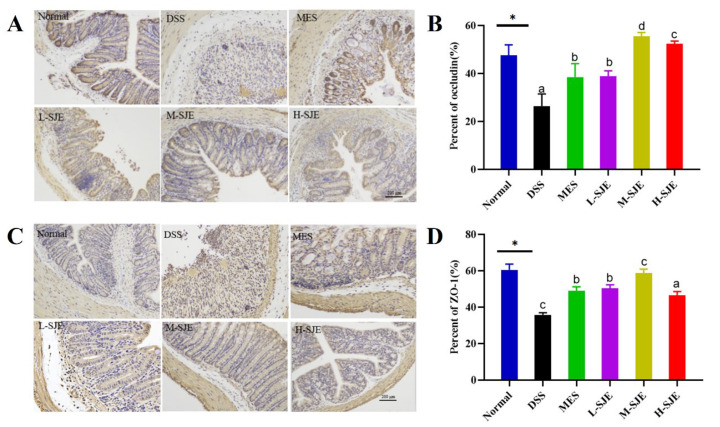
Effects of SJE on gut barrier integrity. Representative pictures of occludin and ZO-1 with amplification (100×). Scale bars, 200 µm. (**A**–**D**) IHC staining and quantitative analysis of occluding and ZO-1. * *p* < 0.05 means statistically significant. Different superscript letters (a–d) in the same graph indicate significant differences between groups (*p* < 0.05).

The protein expression of occludin and ZO-1 was measured, and the results are presented in Figure 3. The normal group was uniformly stained with a large amount of occludin and ZO-1, and the intestinal glands were intact and regular (Figure 3A,C). On the other hand, the DSS treatment decreased the expression levels of occludin and ZO-1 (*p* < 0.05). The oral administration of MES and SJE increased the expression levels of ZO-1 and occludin. In addition, MES and L-SJE exerted similar effects in improving the expression levels of tight junction proteins. Interestingly, a better outcome was achieved after M-SJE treatment (Figure 3B,D).

### 3.4. SJE Regulates Inflammatory Responses in the Colon

**Figure 4:**
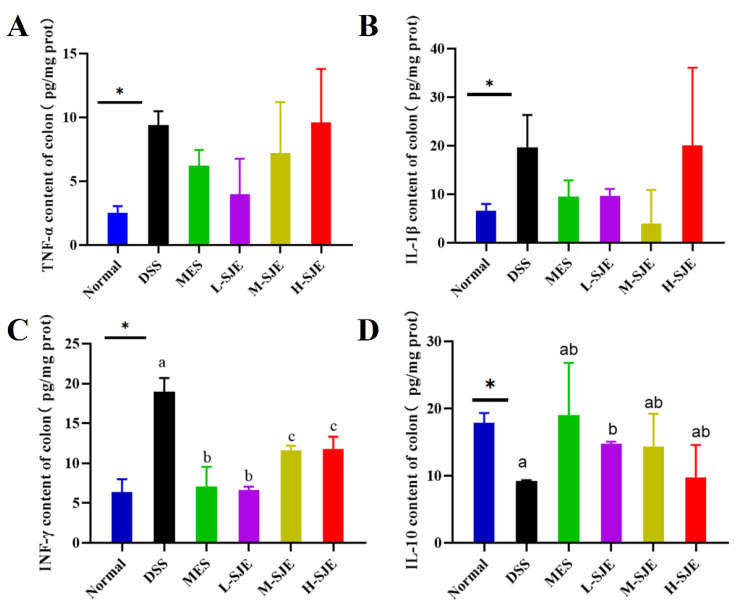
Effects of SJE on inflammatory responses in the colon of DSS-induced colitis mice. The levels of TNF-α (**A**), IL-1β (**B**), INF-γ (**C**) and IL-10 (**D**). * *p* < 0.05 means statistically significant. Different superscript letters (a–c) in the same graph indicate significant differences between groups (*p* < 0.05).

As shown in Figure 4, the DSS treatment significant increased the concentrations of IFN-γ, IL-1β, and TNF-α. However, as for the level of TNF-α and IL-1β, no significant difference was shown among DSS, MES, and the three SJE groups (Figure 4A,B). Interestingly, both MES and SJE treatments decreased the concentration of IFN-γ; however, no statistical difference was observed between MES and L-SJE groups as well as between M-SJE and H-SJE groups (Figure 4C). In addition, compared to the normal group, a remarkable decreasing trend was observed in the anti-inflammatory cytokine IL-10 in the DSS group. Interestingly, L-SJE treatment significant up-regulated the content of IL-10 compared to DSS treatment (Figure 4D).

### 3.5. Measurement of Oxidative Stress Levels in Colonic Tissues

Oxidative stress plays a key role in IBD, and excessive ROS in intestinal tissues leads to an impairment of enzymatic and non-enzymatic antioxidant mechanisms such as SOD and MDA, ultimately causing colonic damage. As shown in Figure S1, the SOD enzyme activity in the M group was significantly decreased compared with the N group (*p* < 0.05), indicating that UC caused a significant imbalance of oxidative stress; the colonic SOD enzyme activity in all subject groups was higher than that in the M group, among which the P, LP and HP groups were significantly higher than that in the M group (*p* < 0.05).

MDA shows the degree of lipid peroxidation, and its content is positively correlated with ROS accumulation. As shown in Figure S1B, the MDA content in group M was significantly higher than that in group N (*p* < 0.05), indicating that UC caused lipid peroxidation; compared with group M, the colon MDA content in all subject groups increased. Compared with the M group, the colon MDA content was reduced in all subject groups, among which P and CHP had the most significant effect (*p* < 0.05). In conclusion, kelp polyphenols can improve the imbalance of oxidative stress caused by UC.

### 3.6. Apoptosis Assay of Colonic Epithelial Cells

In this study, the apoptosis of mouse colon tissue was observed by Tunel staining and the expression of colonic Caspase-3 was observed by immunohistochemical staining to investigate the effect of each subject on the apoptosis of colonic epithelial cells and the possible mechanism of the effect. As shown in Figure S2A, compared with the N group, the M group showed obvious positive staining, indicating the presence of apoptosis in the colonic epithelial cells of UC mice, while the HP group showed no positive staining, indicating that kelp polyphenols could improve apoptosis. As shown in Figure S2B, the positive staining of Caspase-3 was mainly localized in intestinal mucosal epithelial cells and lamina propria inflammatory cell plasma, and the expression was higher in group M. All other subjects inhibited Caspase-3 expression to some extent, among which HP had a better effect. Therefore, the inhibitory effect of kelp polyphenols on an excessive apoptosis of colonic epithelial cells may be achieved through the inhibition of the Caspase-3 apoptotic signaling pathway.

### 3.7. Effect of SJE on Reshaping the Gut Microbiome Structure

**Figure 5:**
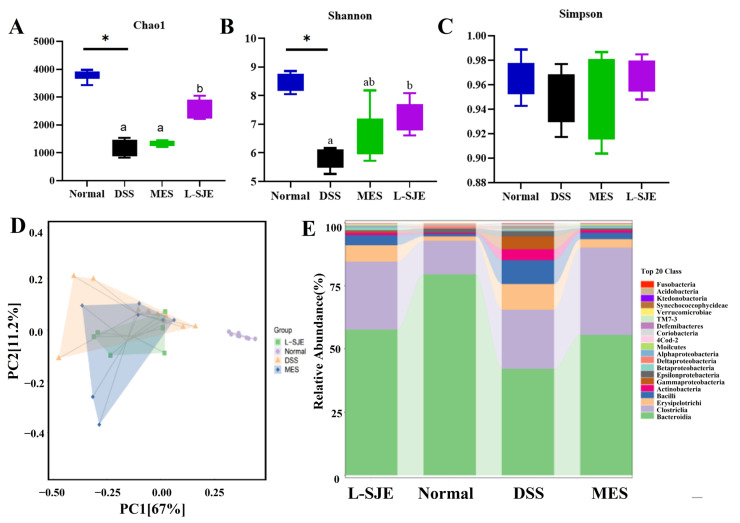
Effects of SJE on gut microbial structure. (**A**) Chao1 index, (**B**) Shannon index, (**C**) Simpson index, (**D**) Principal coordinate analysis (PCA), (**E**) Structure of intestinal microbiota at class level. Different superscript letters (a,b) in the same graph indicate significant differences between groups (*p* < 0.05).

In all other groups compared to the normal group, the Chao1 and Shannon indexes decreased (Figure 5A,B). However, no significant difference was observed between the DSS and MES groups. Interestingly, an oral administration of SJE significantly alleviated the reduction in Chao1 caused by DSS, and the beneficial effects of L-SJE were significant to MES. Compared to the DSS group, MES and L-SJE groups exerted similar effects since an increase in the Shannon indexes was observed. No significant difference was observed between DSS and MES groups. As for the Simpson index, there was no significant difference between these four groups (Figure 5C). The results obtained from the PCA showed gut community composition changes (Figure 5D). The results revealed that the normal and DSS groups had a unique composition in the gut microbiota, while MES and SJE treatments changed gut community composition. 

The structure of intestinal microbiota at the class level is shown in Figure 5E. The results demonstrated that *Bacteroidia*, *Clostridia*, *Erysipelotrichi*, and *Bacilli* were the most abundant microbiota in these four treated groups. Compared to the normal group, DSS treatment significantly reduced the abundance of *Bacteroidia* and raised the relative abundance of *Erysipelotrichi*, *Bacilli*, *Actinobacteria*, and *Gammaproteobacteria*. On the other hand, MES and L-SJE treatments significantly decreased the relative abundance of *Gammaproteobacteria*, *Actinobacteria*, *Bacilli*, and *Erysipelotrichi* compared to the DSS treatment. 

**Figure 6:**
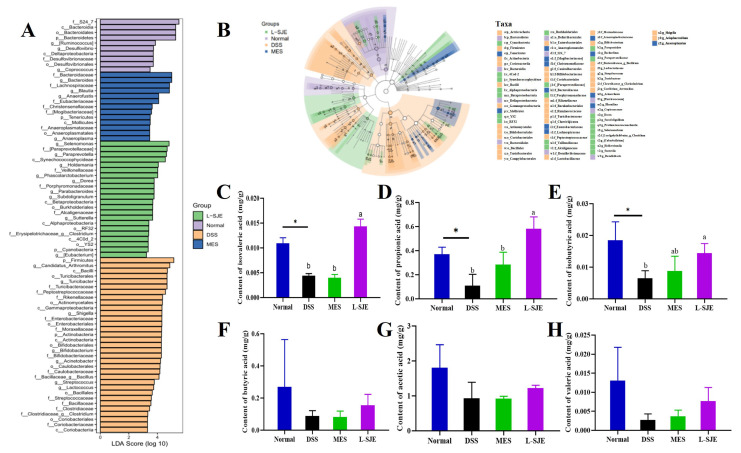
Changes of gut microbiota after different pretreatments and effects of SJE on the production of SCFAs in feces. (**A**) LDA score. (**B**) LEfSe taxonomic cladogram showed key bacterial alterations. (**C**) Content of isovaleric acid, (**D**) propionate acid, (**E**) isobutyric acid, (**F**) butyrate acid, (**G**) acetic acid, and (**H**) valeric acid. Different superscript letters (a,b) in the same graph indicate significant differences between groups (*p* < 0.05).

A linear discriminant analysis (LDA) was used in this study to reveal the statistically different abundant taxons. Different species were observed in these four groups (normal, DSS, MES, and L-SJE). As the LDA score increases (>3), the relative abundance of the corresponding group also increases (Figure 6A). An LEfSe analysis (Figure 6B) was also used in this study, and the results showed changes in key gut microbiota between different groups. The colors of green, purple, yellow, and blue represent the L-SJE, normal, DSS, and MES groups, respectively. The size of the circles presented the relative abundance.

### 3.8. SJE Treatment Increases the SCFAs Content

The effects of SJE on SCFAs in feces are shown in Figure 6. The contents of isovaleric acid (Figure 6C), propionate acid (Figure 6D), and isobutyric acid (Figure 6E) in the DSS group were significantly lower than in the normal group (*p* < 0.05). Interestingly, compared to the other groups, only L-SJE treatment showed significant effects in improving the content of isovaleric acid, propionate acid, and isobutyric acid. On the other hand, no significant beneficial effect was observed after MES treatment. Furthermore, no statistical difference was observed for butyric acid, acetic acid (Figure 6F), and valeric acid (Figure 6H) between all groups.

## 4. Discussion

Many functional foods or dietary components have been evaluated for UC treatment. However, several previous studies focused on the effects of single food components and specific nutrients on UC. Therefore, the protective effects of crude extract and full-nutrient foods on UC treatment remains unclear, and it is necessary to conduct more studies to explore the potential benefits. SJE is rich in polyphenols (about 10%), cellulose, polysaccharides, fatty acids, alginic acid and carotenoids, and it has a strong ability to inhibit oxidase activity and scavenge free radicals with potential efficacy in the treatment of colitis. DSS is a chemical widely used to induce colitis. DSS-induced colitis represents a well-established model to study the pathogenesis of UC and resembles human UC [24]. In the present study, we explored the effects of SJE on DSS-induced colitis. The results showed that orally administered SJE induced several beneficial effects. As shown by colonic alyssum blue staining and HE staining, the DSS group showed massive destruction of cupped cells, inflammatory infiltration, crypt atrophy, and almost disappearance of mucus, kelp polyphenols had a significant protective effect on colonic tissues (*p* < 0.05), and group H showed the best improvement of histological damage in mice: an improved intestinal barrier by up-regulating the expressions of tight junctions (TJs). Interestingly, M-SJE had the strongest recovery effect on tight junction proteins; in general, the functional components exhibit a dose-dependent relationship within an appropriate range. In addition, over a wider dose range, the component may not exhibit an increase in efficacy with increasing dose, meliorated inflammatory responses by promoting anti-inflammatory cytokines and inhibiting the production of pro-inflammatory cytokines, decreased oxidative stress, and regulated intestinal microbial metabolism.

It has been described that the intestinal barrier is a key therapeutic target for UC. Intestinal barrier damage and the abnormal expression of TJs have been found in UC patients [25]. Therefore, an increase in intestinal permeability can accelerate the pathogenesis of this disease, resulting in chronic inflammation. TJs are dynamic multi-protein complexes, which comprise several transmembrane proteins (including occludin and claudin) and scaffolding proteins (including ZO-1 and cingulin). Previous research focusing on the effects of *Laminaria japonica,* also known as SJE, on TJs-related proteins showed that SJE significantly prevented the inhibition of occludin in lipopolysaccharide-stimulated Caco-2 cells (Yang et al., 2019). Consistent with the previous literature, the results showed that the oral administration of SJE notably suppressed the decrease in occludin and ZO-1, suggesting that SJE potentially improved the histological structure and intestinal integrity by up-regulating the expression levels of TJ-related proteins.

The unbalanced generation of inflammatory cytokines causes damage to the colonic mucosa and tissue damage. The anti-inflammatory effects of seaweed extract have been widely confirmed. Ethanol extract of *Laminaria japonica* decreased the pro-inflammatory cytokines in obesity rat models. Yang and collaborators also revealed that *Laminaria japonica* extract decreased the concentration of nitric oxide and IL-6 in lipopolysaccharide-stimulated Caco-2 cells [16]. To further investigate the anti-inflammatory effects of SJE in UC, the contents of pro-inflammatory factors TNF-α, IL-1β, IFN-γ and anti-inflammatory factor IL-10 in mouse colon tissues were measured, in this study, the DSS group had significantly higher expression levels of TNF-α, IL-1β, and INF-γ. After MES and L-SJE treatments, a decreasing trend was observed in suppressing pro-inflammatory factors, mainly in INF-γ. It is secreted by T lymphocytes and macrophages and is involved in the initiation and regulation of multiple immune responses and plays an important role in the inflammatory response. This result can indicate that SJE treatment attenuated the colonic inflammation induced by DSS. Moreover, a lower level of IL-10 appeared in the DSS group. Interestingly, SJE treatment increased the colonic concentration of IL-10. It has been described that anti-inflammatory cytokines had the capacity of inhibiting the overproduction of pro-inflammatory cytokines. Therefore, the results indicated that SJE treatment could attenuate the severity of colon mucosa injury by regulating the inflammatory response. 

It has been demonstrated that apoptosis of colonic epithelial cells is significantly increased in UC disease, which leads to a disruption of colonic integrity, impairment of intestinal mucosal barrier, increased permeability, and the entry of bacteria and other harmful molecules into the intestine to stimulate the release of inflammatory factors and aggravate intestinal injury [26]. In this study, we found that Caspase-3 positive staining was mainly localized in intestinal mucosal epithelial cells and lamina propria inflammatory cell plasma, with higher expression in the DSS group, and Caspase-3 expression was inhibited to some extent in all SJE groups. Therefore, the inhibitory effect of SJE on excessive apoptosis of colonic epithelial cells may be achieved by inhibiting the Caspase-3 apoptotic signaling pathway.

Many studies have confirmed that intestinal microbiota imbalance is closely related to UC disease [27]. Therefore, a reduction in bacterial diversity has been verified in UC patients and animal models with colitis. Nutritional ingredients have been widely used for the prevention of UC disease [28]. A diet rich in dietary fiber and DHA/EPA could up-regulate the proportion of beneficial bacteria and down-regulate harmful bacteria [20,29]. Consistent with previous studies, the DSS treatment significantly decreased the microbial abundance and diversity, and a higher abundance of *Bacilli* and *Gammaproteobacteria* was found in the DSS group [17]. In this experiment, there were 20 species with significant differences in abundance in the DSS group, and the main marker genera were Erysipelotrichaceae, Peptostreptococcaceae, Actinobacteria, Bifidobacterium, Candidatus, Actinobacteria, Bifidobacterium, Candidatus and so on. Among them, Actinobacteria belong to pathogenic bacteria, which can cause a variety of disease infections. Species with significant differences in abundance in the SJE group were the SCFA-producing bacteria Phascolarctobacterium, Butyricimonas, Adlercreutzia, RF32, which was positively associated with quality of life in CD patients, Rikenellaceae, which was significantly reduced in IBD, and Porphyromonadaceae, whose relative abundance was inversely correlated with UC-associated elastase activity [30].

The indigestible nutrients enter the large intestine and are metabolized by the intestinal microbiota to SCFAs and bile acids. The dominant microbiota of different metabolic substrates is significantly different, affecting the structure of the intestinal microbiota [31]. SJE intervention significantly ameliorated the reduction in short-chain fatty acid producing strains such as sacteroidetes, Firmicutes and Proteobacteria caused by DSS. SCFAs, such as acetate, propionate, and butyrate, are the main metabolic products of anaerobic bacteria. Several studies verified the anti-inflammatory function of SCFAs by regulating the generation of TNF-α and IL-10 [32]. In our present study, the SCFAs content obviously decreased after seven days of the DSS treatment. On the other hand, the SJE treatment increased the content of SCFAs, mainly isovaleric, propionate, and isobutyric. Moreover, the results also demonstrated that the inflammatory cytokine IL-10 significantly increased in the SJE groups. Therefore, the oral administration of SJE appeared to relieve intestinal inflammation through regulating intestinal microbiota and SCFAs.

## 5. Conclusions

Altogether, our data demonstrated that the oral administration of SJE prevented the increase in DAI and relieved colonic mucosal damage. The study also showed that SJE treatment presented excellent beneficial effects in regulating the intestinal physical barrier, inhibiting apoptosis, enhancing the level of anti-inflammatory factors, and attenuating oxidative stress. Furthermore, the results revealed that SJE treatment could reshape the colon structure and regulate the metabolism of the intestinal microbiota by increasing its diversity, reducing the proportion of harmful bacteria, including *Bacilli* and *Gammaproteobacteria.* The results could provide a new concept for the nutritional intervention of UC; the SJE can be used as a potential drug for the treatment of UC in humans. Furthermore, all these results have a great significance and could, ultimately, help to improve patients’ quality of life.

## Data Availability

Data is contained within the article or supplementary material.

## Supplementary Materials

The following supporting information can be downloaded at: https://www.mdpi.com/article/10.3390/foods12081671/s1. Figure S1: The activity of SOD (A) and the content of MDA (B) in colon; Figure S2: Effects of SJE on apoptosis of colonic epithelial cells. (A) The IHC representative image of caspase 3scale bars, 200 µm. (B) Tunel staining representative images, scale bars, 100 µm. (C) Caspase 3 histological score. (D) Apoptotic index of tunel staining.
