# Supplementary material for: Saccharina japonica Ethanol Extract Ameliorates Dextran Sulfate Sodium-Induced Colitis via Reshaping Intestinal Microenvironment and Alleviating Inflammatory Response
Source: Foods. 2023 Apr 17;12(8):1671. doi: 10.3390/foods12081671 (PMC10138103; doi:10.3390/foods12081671)
Supplement: Supplementary file 1 [file foods-12-01671-s001.zip › foods-2271710-supplementary.pdf]

Saccharina japonica Ethanol Extract Ameliorates Dextran Sulfate Sodium-Induced Colitis Via Reshaping Intestinal Microenvironment and Alleviating Inflammatory Response

Kuan Lu [1,5], Lin Liu [1], Pengcheng Lin [1], Xiufang Dong [1,3,6], Laixue Ni [6], Hongxia Che [1,3,*] and Wancui Xie [1,2,3,4,5,*]

[1] College of Marine Science and Biological Engineering, Qingdao University of Science and Technology, Qingdao 266042, China
[2] College of Food Science and Engineering, Qingdao Agricultural University, Qingdao 266109, China
[3] Qingdao Special Food Research Institute, Qingdao 266109, China
[4] Shandong Provincial Key Laboratory of Biochemical Engineering, Qingdao 266042, China
[5] Qingdao Keda Future Biotechnology Co., Ltd., Qingdao 266042, China
[6] Linyi Jinluo Wenrui Food Co., Linyi 276007, China
* Correspondence: 03136@qust.edu.cn

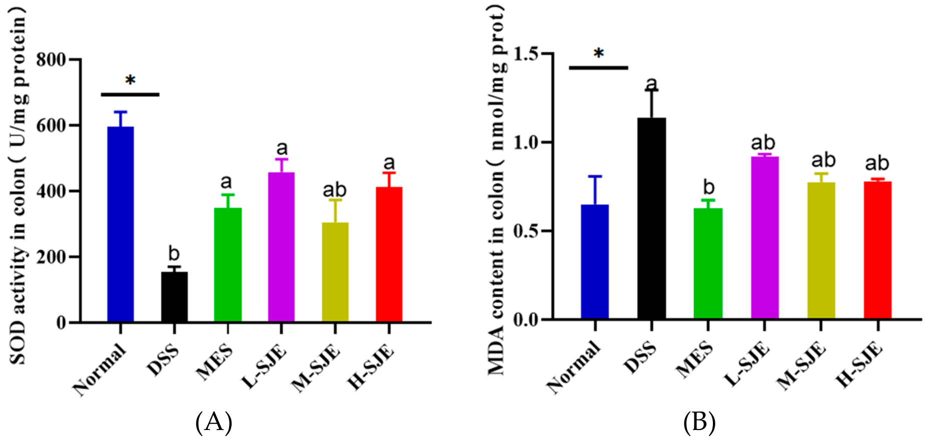

Figure S1. The activity of SOD (A) and the content of MDA (B) in colon

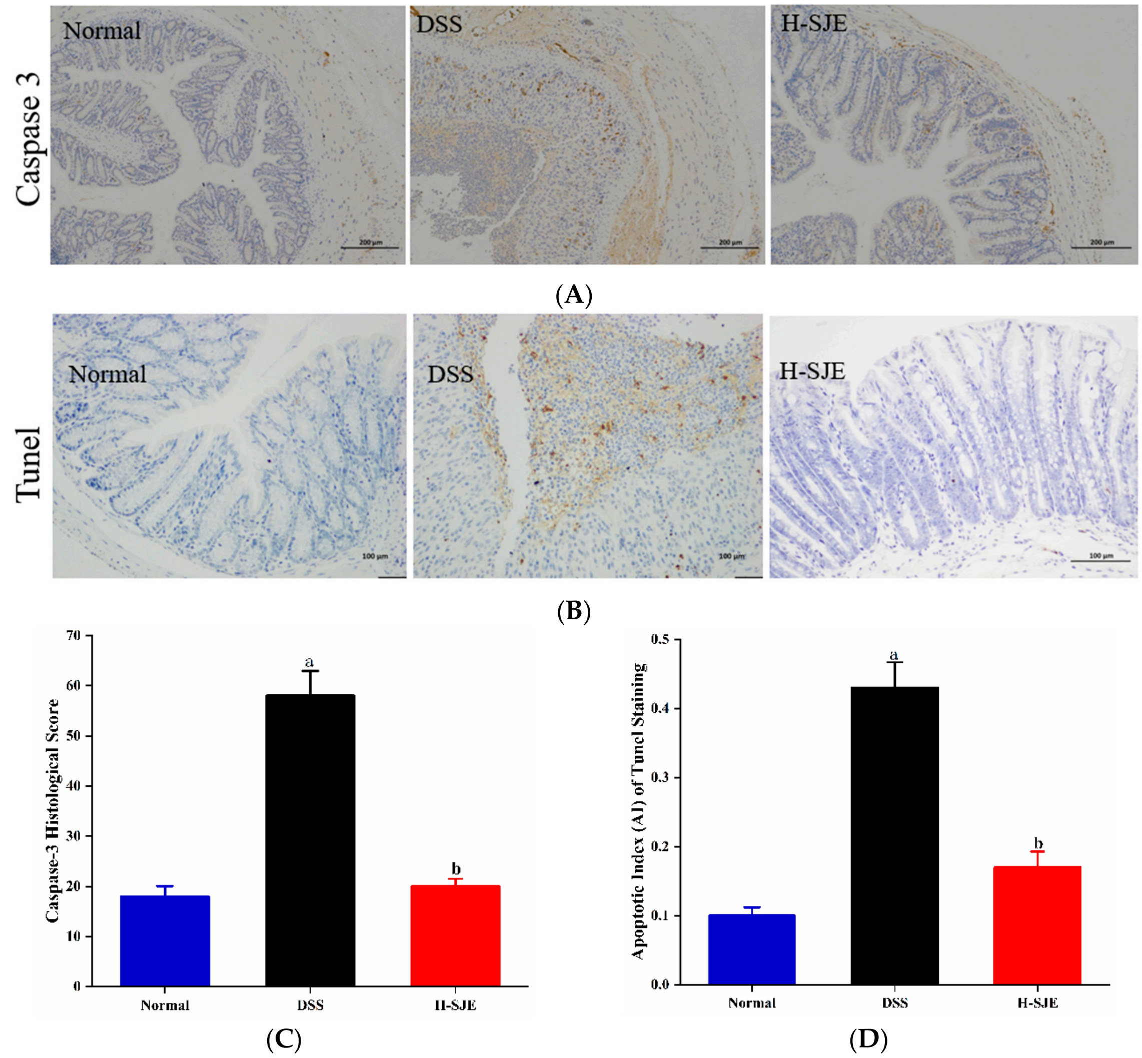

Figure S2. Effects of SJE on apoptosis of colonic epithelial cells. (A) The IHC representative image of caspase 3scale bars, 200 µm. (B) Tunel staining representative images, scale bars, 100 µm. (C) Caspase 3 histological score. (D) Apoptotic index of tunel staining.
